# Commentary - Radiology in India: The Next Decade

**DOI:** 10.4103/0971-3026.41869

**Published:** 2008-08

**Authors:** Arjun Kalyanpur

**Affiliations:** Chief Radiologist, Teleradiology Solutions, 7G, Opp. Graphite India, Whitefield, Bangalore, India. Email: arjun.kalyanpur@telradsol.com

‘It is not because things are difficult that we do not dare; it is because we do not dare that they are difficult’

- Seneca

My personal view is that the next decade will be a critical and, at the same time, an exciting one for Indian radiology, with major challenges and equally major opportunities.

India is a country with a billion people and fewer than 10,000 radiologists, a severely imbalanced ratio of 1:100,000 (the corresponding ratio in the US is 1:10,000). The challenge for academic radiology in India is therefore going to be the problem of educating and training a sufficient number of residents, while coping with increasingly busy clinical practices. This will necessitate more training programs across the country, with clinical/practicing radiologists needing to play a more active role in training young residents. Private imaging centers and nongovernmental institutions will play an increasingly larger role in training the next generation of radiologists, as academic centers will be unable to meet the demands in their entirety.

At the same time, there will need to be a change in the training focus to allow a more broad-based learning to keep pace with the new developments in modern imaging. It will be necessary for the training programs to provide training and experience in all contemporary branches of radiology, with commensurate (if not equal) importance given to specializations/modalities like breast imaging and cardiovascular imaging as is currently given to conventional radiology. This is not to imply by any means that we should abandon our traditional strength in conventional radiology. (In fact, training programs in the West have succeeded in placing considerable emphasis on conventional imaging while simultaneously focusing on the modern modalities.) I also believe that there will need to be a paradigm shift in the examination standard - from expecting encyclopedic perfection to requiring general and basic competence: we need radiologists who are safe, logical, and competent.

In terms of technology, the trend over the next decade, as we have seen over the last one, will continue to be the rapid adoption of newer technologies such as digital radiography, mammography, high-end, multidetector row CT scan, and high-field strength MRI. The technology gap between the West and the East will continue to narrow, with newer technologies emerging in India contemporaneously with their launch in the Western world.

With larger volumes of radiologic studies to be interpreted, there will be an increasing demand for technologies to make radiologists more efficient; for example, picture archival and communications systems (PACS) and radiology information systems (RIS). Implementation of PACS and RIS has been seen to increase efficiency and productivity in radiology departments by up to 50% and to reduce examination times to a commensurate degree. Similarly, newer digital technologies such as voice recognition and structured formatted reporting significantly enhance radiologists' workflow, productivity, and reporting accuracy. I foresee more and more imaging departments across the country recognizing the need to embrace digital technologies over the next decade, and benefiting greatly from this.

Teleradiology, a subject close to my own heart, will, over the next decade, be adopted throughout India in increasing measure, allowing for three changes: greater reach of delivery of high-quality radiologic services to remote locations, increasing subspecialization within the field of radiology, and increasing provision of emergency radiology services. The cost of technology (both software and storage hardware) will progressively decrease in keeping with Moore's law and the speed of telecommunications will continuously rise to allow for faster and more efficient data transfer. Rural India will benefit from this heartening trend. In addition, teleradiology will continue to keep Indian radiology in the limelight on the global stage.

Screening radiography, as opposed to diagnostic radiography, will play an ever-increasing role in imaging utilization. Whether it be USG for detecting pelvic malignancies, mammography for breast cancer, or coronary CT angiography (CTA) for coronary artery disease, all of these will continue to evolve and become entrenched in their respective niches. Given that India has among the highest rates of breast cancer mortality in the world and that breast cancer is curable if detected early, a comprehensive breast screening program is clearly of national importance. Similarly, the high prevalence of coronary artery disease in India, coupled with its higher and earlier mortality in our country, presents a compelling argument for greater utilization of a screening test such as coronary CTA that is both sensitive and specific, unlike its noninvasive precursors.

On the flip side of the screening coin is the issue of radiation dose, which, over the next decade, will need to be taken with the utmost seriousness by radiologists in India. We are often careless of the impact and consequences of radiation, and the controversy that is erupting on the other side of the world should serve as a reminder to us of the critical importance of limiting radiation dose as far as possible while practicing our specialty. We owe this to our patients and to the community at large.

Overwhelmingly, the need of the hour, and what will truly impact on our performance as radiologists over the next decade will be a heightened awareness on our part of quality assurance parameters in radiology; this includes implementation of interpretation standards, standardized imaging protocols, communication and security standards, periodic assessment of quality and, overall, a willingness to allow our errors to be exposed in an effort to improve our performance and the benefit to our patients. While we may be concerned that this may expose us to legal risk in the short term, in the long term this will benefit us, increase our credibility among our clinical colleagues and inevitably strengthen our overall abilities as radiologists.

In this regard, continuing medical education programs for practicing radiologists, which are now routine in the West, represent an idea whose time has come in India as well. An increasing number of such programs have emerged over the previous few years in our country and is the harbinger of a bright future.

We are on the verge of entering a new and inspirational era in diagnostic imaging, one driven by digital radiology and powered by the internet and information technology, in which global barriers are falling. Our challenge in India is to retain the best of the old even as we embrace the new. Today, I feel that the main obstacles to our progress lie only within ourselves.

